# Characterization of MRSA and ESBL pathogens from patients with surgical-site infections in Accra, Ghana

**DOI:** 10.1017/ash.2022.219

**Published:** 2022-05-16

**Authors:** Terrel Sanders, Jeannette Bentum, Anne Fox, Beverly Egyir, Chaselynn Watters

## Abstract

**Background:** In Ghana, treatment of surgical site infections (SSIs) is often empirical and not based on targeted therapy (ie, knowledge of the organisms infecting surgical sites or their susceptibility profiles). This empirical approach most often leads to inappropriate prescription, which is a major driver of antimicrobial resistance. Using phenotypic and molecular tools, we investigated *S. aureus*, *E. coli*, *K. pneumoniae*, and *P. aeruginosa* recovered from patients with SSIs. **Methods:** Identification of bacteria species recovered from wound swabs and aspirates was performed using matrix-assisted laser desorption/ionization time-of-flight mass spectroscopy (MALDI-TOF MS). Antimicrobial susceptibility testing (AST) was done using the Kirby-Bauer disk diffusion method. Results were interpreted according to the CLSI 2018 guidelines. Extended-spectrum β-lactamase (ESBL) positivity was detected among the gram-negative isolates using the double disk-diffusion method and PCR amplification of ESBL genes (*bla*SHV, *bla*TEM, and *bla*CTX-M). *Staphylococcus aureus* isolates resistant to cefoxitin were further tested for the presence of *mecA* using PCR. **Results:** In total, 312 patients were enrolled in this prospective study. The 243 bacteria species identified comprised *Escherichia coli* (34%; 107 of 312), *Klebsiella pneumoniae* (20%; 62 of 312), *Pseudomonas aeruginosa* (16%; 49 of 312), and *S. aureus* (8%; 25 of 312). *S. aureus* isolates were susceptible to clindamycin, erythromycin, gentamicin, linezolid, rifampicin, and norfloxacin, but 10 *S. aureus* isolates were resistant to cefoxitin and were positive for the *mecA* gene (MRSA). Among the 169 isolates in the Enterobactericae category (*E. coli* and *K. pneumoniae*), 143 (85%) were resistant to tetracycline; 141 (83%) were resistant to trimethoprim–sulfamethoxazole; 118 (70%) were resistant to cefotaxime; 111 (66%) were resistant to cefuroxime; 98 (58%) were resistant to ciprofloxacin; 86 (51%) were resistant to gentamicin; and 81 (48%) were resistant to chloramphenicol. However, 161 (95%) were sensitive to amikacin and 159 (94%) were sensitive to meropenem. Among the 49 *P. aeruginosa* isolates, 45 (92%) showed sensitivity to amikacin, 43 (88%) showed sensitivity to meropenem, 35 (71%) showed sensitivity to gentamicin, and 35 (71%) showed sensitivity to ciprofloxacin. ESBL was detected in 59 (55%) of 107 *E. coli* isolates, and 48 (77%) of 62 *K. pneumoniae* isolates. *bla*CTX-M was the dominant ESBL gene in *E. coli* isolates (34 of 59, 58%). For *K. pneumoniae* isolates, *bla*CTX-M genes were detected in 45 (94%) of 48 isolates and *bla*SHV genes were detected in 44 (92%) of 48 isolates. Among the 49 *P. aeruginosa* isolates, 3 harbored the *bla*TEM gene. **Conclusions:** The findings of high proportions of ESBL-producing bacteria species in Ghana is a grave public health concern. Data generated in this study will inform treatment decisions and policies and appropriate antibiogram development and will support antimicrobial stewardship programs at the respective healthcare facilities in Ghana.

**Funding:** None

**Disclosures:** None

